# “I am proud to be a midwife because I save a lot of lives”: survey and interviews with graduates of International Medical Corps’ midwifery education programme in South Sudan

**DOI:** 10.1080/26410397.2026.2694248

**Published:** 2026-07-31

**Authors:** Sara E. Casey, Guma Patrick Isa, Abdou Sebushishe, Preethika Sundararaj, Megan Piccirillo, Javed Ali, Shiromi M. Perera

**Affiliations:** a Associate Professor, RAISE Initiative, Heilbrunn Department of Population and Family Health, Mailman School of Public Health, Columbia University, New York, NY, USA. *Correspondence*: sec42@columbia.edu; b M&E Coordinator, International Medical Corps, Juba, South Sudan; c Medical Director, International Medical Corps, Juba, South Sudan; d Research Assistant, RAISE Initiative, Heilbrunn Department of Population and Family Health, Mailman School of Public Health, Columbia University, New York, NY, USA; e Student research assistant, RAISE Initiative, Heilbrunn Department of Population and Family Health, Mailman School of Public Health, Columbia University, New York, NY, USA; f Senior Director, Emergency Response Unit, International Medical Corps, Washington DC, USA; g Senior Research Specialist, Research, Evidence and Learning Unit, International Medical Corps, Washington DC, USA

**Keywords:** midwives, midwifery education, South Sudan, sexual and reproductive health, armed conflict, humanitarian

## Abstract

South Sudan has extremely high maternal mortality, driven, in part, by an underfunded health system and a shortage of trained health workers. Skilled birth attendants are essential to reducing maternal mortality. International Medical Corps supports competency-based midwifery education in three schools in South Sudan, offering a 2.5-year certificate and a 3-year diploma aligned with international standards for midwifery practice. A mixed-methods evaluation was conducted to assess the strengths and challenges of the midwifery education programme to inform programme improvement. This manuscript presents data on midwife graduates’ perspectives on the training and their work as midwives. A survey was conducted with 314 graduates, complemented by in-depth interviews (n = 15) and daily task diaries (n = 21). Most graduates (76.4%) were currently working as midwives, with nearly half (47.5%) based in rural areas. They reported high satisfaction with their training, felt largely prepared for their roles and expressed pride in their profession. Graduates felt most competent in providing antenatal care, safe childbirth services and short-acting contraceptives and least competent in providing prevention of mother-to-child transmission of HIV and long-acting reversible contraceptives. Commonly reported challenges included inadequate infrastructure and insecurity. Given the limited evidence on midwifery graduates’ experiences in conflict-affected settings, these findings offer valuable insights for strengthening midwifery education and workforce support. Graduates work throughout the country, including in rural areas, supporting women through safe and healthy pregnancies. Strong partnerships between ministries of health and partners are essential to address challenges, support midwives and strengthen health systems, while upholding women’s rights to good quality, respectful care.

## Plain language summary

South Sudan has the highest number of maternal deaths in the world. This is partly because the health system is underfunded and there are not enough trained health workers. Skilled birth attendants, like midwives, play a key role in reducing maternal deaths. International Medical Corps supported three midwifery schools in the country to help address the shortage. This manuscript shares findings from our assessment of the midwifery education programme. We carried out a survey of 314 graduates of the three schools. We also conducted 15 interviews and looked at 21 daily task diaries from a smaller group of graduates. We found that most graduates (76.4%) were currently working as midwives. Nearly half (47.5%) were based in rural areas. Overall, graduates reported the quality of the education received was good or very good, and felt well-prepared for work as a midwife. They felt most confident in providing antenatal care, normal childbirth care and short-acting contraceptive methods. They felt least confident in providing prevention of mother-to-child transmission of HIV services and long-acting reversible contraceptives. Inadequate infrastructure and medical supplies and insecurity were frequently mentioned challenges. Midwives are serving communities across South Sudan, particularly in rural areas. They support safe and healthy pregnancies and help women and couples to exercise their reproductive rights. Strong partnerships among ministries of health and partners in fragile settings are essential. They should support midwives’ professional development and strengthen health systems. This upholds women’s rights to good quality, respectful sexual and reproductive health care.

## Résumé

Le Soudan du Sud enregistre un taux de mortalité maternelle extrêmement élevé, dû en partie au sous-financement du système de santé et à une pénurie de personnel de santé qualifié. L’assistance de sages-femmes compétentes est essentielle pour réduire la mortalité maternelle. L’International Medical Corps soutient une formation de sage-femme axée sur les compétences dans trois écoles du Soudan du Sud, proposant un certificat en deux ans et demi et un diplôme en trois ans conformes aux normes internationales pour la pratique de la profession de sage-femme. Une évaluation à méthodes mixtes a été réalisée pour évaluer les forces et les faiblesses du programme de formation et guider son amélioration. Cet article présente des données sur le point de vue des sages-femmes diplômées concernant leur formation et leur travail. Une enquête a été menée auprès de 314 diplômées, complétée par des entretiens approfondis (n = 15) et la tenue de journaux de bord quotidiens (n = 21). La plupart des diplômées (76,4%) exerçaient la profession de sage-femme, dont près de la moitié (47,5%) en zone rurale. Elles se sont déclarées très satisfaites de leur formation, se sentaient bien préparées à leur rôle et étaient fières de leur métier. Les diplômées se sentaient particulièrement compétentes pour assurer les soins prénatals, les accouchements sans risque et la contraception de courte durée, et moins compétentes pour la prévention de la transmission du VIH de la mère à l’enfant et la contraception réversible de longue durée. Parmi les difficultés fréquemment citées figuraient l’insuffisance des infrastructures et l’insécurité. Compte tenu de la rareté des données disponibles sur l’expérience des sages-femmes diplômées dans des environnements touchés par les conflits, ces résultats offrent un éclairage précieux pour renforcer la formation des sages-femmes et le soutien apporté aux personnels. Les diplômées exercent dans tout le pays, notamment dans les zones rurales, et elles accompagnent les femmes tout au long de grossesses saines et sécurisées. De solides collaborations entre les ministères de la santé et les partenaires sont essentielles pour relever les défis, épauler les sages-femmes et consolider les systèmes de santé, tout en garantissant le droit des femmes à des soins respectueux et de qualité.

## Resumen

Sudán del Sur tiene una tasa de mortalidad materna sumamente elevada, impulsada en parte por un sistema de salud subfinanciado y una escasez de trabajadores de salud calificados. Las asistentes de parto calificadas son esenciales para reducir la mortalidad materna. *International Medical Corps* apoya la educación en partería basada en competencias en tres escuelas de Sudán del Sur; ofrece un certificado de 2.5 años y un diploma de 3 años alineados con las normas internacionales del ejercicio de la partería. Se realizó una evaluación de métodos mixtos para evaluar las fortalezas y los retos del programa de educación en partería, con el fin de informar el mejoramiento del programa. Este manuscrito presenta datos sobre las perspectivas de las graduadas de partería sobre su formación y su trabajo como parteras. Se realizó una encuesta con 314 graduadas, que fue suplementada con entrevistas a profundidad (n = 15) y diarios de tareas cotidianas (n = 21). La mayoría de las graduadas (76.4%) estaban trabajando como parteras, y casi la mitad (47.5%) residía en zonas rurales. Informaron una alta satisfacción con su formación, se sentían muy preparadas para desempeñar sus funciones y expresaron orgullo por su profesión. Las graduadas se sentían más competentes en brindar atención prenatal, prestar servicios de parto seguro y proporcionar anticonceptivos de corta duración, y menos competentes en proporcionar prevención de la transmisión materno-infantil del VIH y anticonceptivos reversibles de acción prolongada. Entre los retos reportados comúnmente figuraban una infraestructura inadecuada e inseguridad. En vista de la evidencia limitada sobre las experiencias de las graduadas de partería en entornos afectados por conflictos, estos hallazgos ofrecen perspectivas valiosas para fortalecer la educación en partería y el apoyo a la fuerza laboral. Las graduadas trabajan por todo el país, incluso en zonas rurales, apoyando a las mujeres para tener embarazos seguros y sanos. Las alianzas sólidas entre ministerios de salud y socios son esenciales para abordar retos, apoyar a las parteras y fortalecer los sistemas de salud, a la vez que se defiende el derecho de las mujeres de recibir una atención respetuosa y de buena calidad.

## Introduction

South Sudan has one of the highest maternal mortality ratios (MMR) in the world. The MMR has declined from 1687 to 1223 maternal deaths per 100,000 live births since 2000,^1^ largely due to the increase in the number of qualified midwives. According to the South Sudan Nurses and Midwives Association (SSNAMA), the number increased from just 12 at the time of independence in 2011 to approximately 1500 in 2024.^2^ Despite this progress, pregnancy and childbirth complications remain the leading causes of death among women and girls, largely due to the limited availability of quality obstetric services and skilled birth attendants. In South Sudan, the vast majority of births (87%) occur at home, and fewer than 20% are attended by trained health workers.^3,4^ Neonatal mortality is also high at 40 per 1000 live births.^5^ Only 37% of pregnant women attend four or more antenatal care visits.^6^ Numerous factors contribute to these poor health outcomes, including an underfunded healthcare system (with less than 2% of the annual national budget allocated to health), severe shortages in human resources for health (one midwife per 39,088 people compared to WHO recommendation of 30 per 10,000), low numbers of functional health facilities offering maternal health services, poor facility infrastructure, limited access to health care, conflict, cultural and gender norms, low levels of education, and the costs associated with accessing services.^4,7^

Skilled birth attendance, such as by a qualified midwife, can help reverse these trends and significantly reduce maternal and neonatal mortality.^8^ UNFPA and WHO report that with the proper investment, midwives could save millions of lives globally each year, averting roughly two-thirds of maternal and newborn deaths.^9,10^ When educated, licenced and integrated into interdisciplinary teams within supportive environments with necessary supplies and equipment, midwives are associated with a wide range of positive outcomes, from improved health indicators to broader societal benefits, including economic stabilisation.^10^ Midwives are trained to deliver comprehensive sexual and reproductive health (SRH) information and services, promote community health, and empower women and couples to exercise their reproductive rights through culturally sensitive care.^11^ Expanding and strengthening midwifery education and workforce capacity is, therefore, essential not only to improving health outcomes but also to realising sexual and reproductive health and rights. Ensuring that midwives are competent, well-supported, and protected upholds women’s rights to health, bodily autonomy, and respectful maternity care and advances global commitments to gender equality and the Sustainable Development Goals.^12–14^

Despite the many benefits and vital role of midwives, they are in critical shortage in many low- and middle- income countries. This is in large part due to difficult, unsupportive working conditions and meagre salaries.^11,15^ In South Sudan, decades of conflict and ensuing instability have contributed to this shortage by severely impacting the public health infrastructure, especially in rural areas of the country, where an estimated 79% of the population reside,^16–18^ and less than half (44%) live within five kilometres of a functional health facility.^19^ Protracted conflict has damaged health facilities, caused periodic closures, disrupted supply chains, and displaced both communities and health workers, further weakening service delivery. Insecurity and population displacement limit access to antenatal, delivery, and emergency obstetric care, impacting maternal and newborn health.^17,20^ An analysis of data from 181 countries found that countries affected by war have mean MMRs double the MMRs in conflict-free countries.^21^ Concerns over the security and safety of midwives in South Sudan remain a significant challenge, affecting recruitment and retention. Insecurity has undermined the deployment of midwives to rural areas mostly due to a lack of incentives and support strategies.^22^ Some midwives are even afraid to attend births at night in their own communities due to fear of attack.^15^

International Medical Corps (IMC) was among the first organisations to support midwifery education in South Sudan, contributing to the rapid increase in the number of midwives nationally. Since 2013, IMC has co-managed and supported three midwifery schools: Juba College of Nursing and Midwifery (JCONAM), Kajo Keji Health Sciences Institute (KKHSI) and Wau Health Sciences Institute (WHSI), offering diploma and certificate programmes in midwifery. The 3-year diploma, or “registered midwifery” programme, meets the standards established by the International Confederation of Midwives (ICM) on essential competencies,^23^ including research, management and leadership training. The “enrolled midwifery” programme is a 2.5-year certificate programme, with similar clinical content but excluding the research, management, and leadership components. Students’ classroom learning is supplemented with demonstrations and practice sessions in a skills laboratory prior to their clinical practicum. Clinical practice takes place at nearby hospitals where students gain exposure to medical, surgical, paediatric, and obstetric/gynecology units as well as at Primary Health Care Centres (PHCC) to gain experience in primary care and community-level health services. As of early 2024, nearly 500 midwives had graduated from the three schools.

In 2022, IMC, in collaboration with the Reproductive Health Access, Information and Services in Emergencies (RAISE) Initiative at Columbia University, conducted a cross-sectional mixed-methods evaluation to determine the strengths and challenges of IMC’s midwifery education programme across three schools to inform programme improvement. A mixed-methods design, including rapid assessments of the schools; interviews with faculty, graduates and current supervisors; daily task registers and focus group discussions with female users of midwifery care, was used to provide a holistic understanding of the midwifery education programme across multiple stakeholders. This manuscript presents data from graduates of the three programmes; results from other stakeholders and components are published elsewhere.^24,25^

## Methods

This manuscript presents findings only from midwifery school graduates, a critical source of data as beneficiaries of the education programme. The objectives of this component of the overall evaluation were to determine graduates’ employment after graduation; their perceptions of the programme’s strengths and weaknesses in preparing them for professional practice and recommendations for improvement; their perceived competence providing SRH services (e.g. self-reported ability to perform specific skills) and the frequency with which they provide SRH services, as well as their experiences and the challenges they face in practising as midwives. Data were collected from graduates via a representative survey of graduates (n = 314), complemented by in-depth interviews (IDI) (n = 15) and daily task diaries (n = 21) with subsets of survey respondents. The IDIs were intended to expand on the survey data while the task diaries were used to estimate the amount of time graduates spent practising the skills they learned in their work.

### Study tools

The survey questionnaire was designed for phone administration and included questions on graduates’ socio-demographic characteristics, perceptions of their educational experience, work history since graduation, frequency of and perceived competence in providing various SRH services (Supplementary file 1). The questionnaire was programmed onto tablets using KoboToolbox, with built-in consistency checks to improve data quality. The IDI guide further explored graduates’ perceptions of the strengths and weaknesses of their training and recommendations to improve it, their feelings of preparedness to practise upon graduation, and their experience working as a midwife, including challenges they face (Supplementary file 1). The daily task diary was adapted from tools used previously by Jhpiego.^26^

### Data collection training

Seventeen male and female interviewers and one research manager completed a 5-day training on quantitative methods and research ethics. Following the training, 10 individuals (4 female, 6 male) were selected to conduct survey interviews. Simultaneously, four interviewers, who were themselves midwives (3 female, 1 male), were trained to conduct the IDIs.

### Survey of midwifery graduates

#### Sample

The researchers worked with the administration of the three midwifery schools to compile a list of graduates of the IMC-supported midwifery programme at the three schools that was then supplemented with data from the SSNAMA. A total of 442 students completed the registered or enrolled midwife programmes at the schools from 2013 to 2021. The team invited all graduates for whom contact information was available (n = 348) to participate in the survey.

#### Data collection

Ten interviewers contacted graduates by phone from the IMC Juba office between March 14 and 18, 2022. They explained the survey using an information sheet before obtaining verbal informed consent to participate. Interviews were conducted in English, the language of instruction in the midwifery schools, and data were entered into a tablet. Interviewers asked members of the same cohort to share contact information for those graduates whose contact information was missing or incorrect. Additional efforts were made to reach graduates via email, when available, to request a phone number. One interviewer returned for ten days over two additional weeks to contact graduates who were unavailable during the first round of calls. At the end of each day of data collection, members of the research team reviewed the uploaded data and addressed errors with the interviewers.

#### Data analysis

Survey data were exported to SPSS (v27) for cleaning and descriptive analysis, including frequencies and means. Chi-square tests were used to compare categorical variables by sex; however, no significant differences in the data were found in this comparison. Therefore, the data are presented for all midwives.

### In-depth interviews with midwifery graduates

#### Sample

Fifteen graduates who were working as midwives in or near Juba, Wau or Malakal towns were purposively selected among survey respondents based on graduation dates, schools and gender for IDIs. Potential candidates were called by an interviewer to determine their eligibility, determine accessibility of the research team to their work site, explain the study and obtain consent for a visit. As Kajo Keji was still insecure at the time of the study due to ongoing conflict, Malakal was identified as an alternative site because many graduates were working in that area and IMC had an office there to support data collection.

#### Data collection

Two qualitative research teams of two interviewers each spent a week in Wau or Malakal for data collection, and one team also conducted interviews in Juba. Interviewers explained the evaluation using an information sheet before obtaining verbal informed consent, including to audio record the interview. In-person interviews with graduates were conducted in English and lasted 30–45 minutes.

#### Data analysis

IDIs were transcribed verbatim. Members of the research team listened to a selection of the recordings and compared the transcripts for quality assurance. Further clarifications were made with research team members in South Sudan. Researchers reviewed the transcripts, used an inductive approach to create a codebook with the major themes, and then uploaded them to Dedoose.^27^ After double coding the first two transcripts and then discussing and resolving differences, one researcher coded the remainder, with a second researcher double coding 20% of the transcripts. After coding was completed, researchers used thematic analysis to identify the main themes.

### Daily task diaries

#### Sample

A convenience sample of 21 midwives, who also participated in the survey and worked in 10 IMC-supported health facilities, were asked to record the amount of time they spent on various services and tasks in a daily task diary over a 14-day period.

#### Data collection

After training by the research team, IMC staff obtained verbal informed consent from the midwives they supervised and trained them how to complete the diaries. They emailed scans of the completed diaries to the research team, who reviewed and entered the data into Excel. Research team members routinely clarified any discrepancies or missing data with the IMC staff.

#### Data analysis

Daily task diary data were entered and cleaned in Excel before summarising the time spent on different services and tasks with frequencies and proportions.

### Reflexivity statement

The research team comprised individuals of different genders, professional backgrounds, and geographic origins, including South Sudan, bringing a range of perspectives in public health, SRH, medicine, research, and monitoring and evaluation. This diversity helped balance insider and outsider viewpoints, strengthen cultural relevance, and mitigate individual or disciplinary bias. Team members based in South Sudan played a key role in data collection, contextual interpretation, validation of findings, and the development of recommendations, helping to address power imbalances between international and local researchers. Regular conversations throughout the process fostered critical reflection on assumptions and enhanced the transparency and credibility of the research process. Senior investigators mentored early-career public health researchers, specifically two Master of Public Health students, who brought their perspectives while also building new skills. Given differences in nationality, professional seniority, and institutional affiliation, the team was aware of potential power imbalances between researchers and participants. To mitigate these dynamics, South Sudanese team members conducted all data collection with participants, fostering trust and ensuring participants felt comfortable sharing their experiences. Interactions emphasised voluntary participation, confidentiality, and respectful engagement to minimise the influence of hierarchical or outsider status on the data collected.

### Ethical considerations

The Institutional Review Boards of Columbia University (IRB-AAAT8763, October 10, 2021) and the Ministry of Health (MOH) for the Republic of South Sudan (September 27, 2021, by the Office of the Undersecretary) determined the study to be exempt from approval because participants responded in their professional capacity describing their work and education, and no personal identifying information was collected. Only members of the research team had access to the data. The lists used to contact graduates were destroyed after data collection. All participants were identified with a code number in the data, and no names were recorded.

## Results

Phone interviews were completed with 314 of the 348 graduates of the three midwifery programmes; the remaining 10% could not be reached via phone or email. IDIs were conducted with 15 graduates working as midwives who also completed the survey. Results are presented by study objective and are supplemented with qualitative findings from the interviews and data from 21 graduates’ clinical task diaries capturing 274 total workdays.

### Socio-demographics and education

Nearly half of survey respondents were graduates of KKHSI (46.2%), followed by WHSI (30.9%) and JCONAM (22.9%) (Table 1). Two-thirds (68.2%) completed the 3-year diploma programme, while 31.8% completed the 2.5-year certificate programme. Over 75% graduated since 2016, and 93% received scholarship support from IMC. Over half (59.2%) of the surveyed graduates were female, and they had a mean age of 31.5 years. Three-quarters were currently or previously married and had at least one child. A sizeable minority reported being currently displaced from their home in South Sudan (17.2%) or currently a refugee living outside the country (13.1%).

**Table 1.** Socio-demographics and education

	Survey n = 314 (%)	IDI participants n = 15
**Midwifery school**		
JCONAM	72 (22.9%)	2
KKHSI	145 (46.2%)	8
WHSI	97 (30.9%)	5
**Midwifery programme**		
Registered (3-year diploma) midwife	214 (68.2%)	10
Enrolled (2.5 certificate) midwife	100 (31.8%)	5
**Year of graduation**		
2013–2015	65 (20.7%)	3
2016–2018	127 (40.4%)	8
2019–2021	122 (38.9%)	4
**Received scholarship support from IMC**	292 (93.0%)	15
**Gender**		
Female	186 (59.2%)	6
Male	129 (40.8%)	9
**Mean age (SD) in years**	31.5 (5.1)	
23–30 years	117 (37.3%)	8
30–39 years	174 (55.4%)	7
40 years or older	23 (7.3%)	0
**Marital status**		
Never married	69 (22.0%)	*ND*
Currently or previously married	245 (78.0%)	*ND*
**No. of children**		
None	87 (27.7%)	*ND*
1–2	139 (44.3%)	*ND*
3 or more	88 (28.0%)	*ND*
**Forcibly displaced**		
No	219 (69.7%)	*ND*
Internally displaced person (IDP)	54 (17.2%)	*ND*
Refugee outside South Sudan	41 (13.1%)	*ND*

*ND: No data. These questions were not asked of IDI participants. No sociodemographic data were collected from task diary participants.

Nine of 15 IDI participants were male, and half attended KKHSI (n = 8). Ten graduated from the three-year Registered Midwife programme, and over half graduated between 2016 and 2018. All 15 received scholarship support from IMC. At the time of the interviews, two IDI participants were working in hospitals, 10 in primary healthcare centres, and three in NGO-run health centres in internally displaced persons (IDP) camps.

### Current employment

Most graduates reported finding a job within one year of graduation (Table 2). The majority (76.4%) were working as a midwife in a clinical capacity, with 60.8% in a primary care facility and 35.8% in a hospital. Most (88.7%) worked in health facilities receiving some kind of NGO support. Two-thirds (66.3%) were employed by NGOs or religious institutions and 25.8% by the MOH. Nearly half (47.5%) worked in a rural area and 39.2% in a town, while 13.3% were employed in Juba. Only 21.7% were in their first job post-graduation. The primary reasons reported for leaving previous jobs were being let go due to project closure or loss of funding (29.3%), finding a better job (16.9%), low or irregular pay (14.1%) or a difficult work environment (13.8%). Over half (55.9%) of those who were not working as a midwife were unemployed and looking for work.

**Table 2.** Current employment (n = 314)

	n (%)
**Length of time after graduation before starting 1st job**	
Immediately or within 1 month	100 (31.9%)
Less than 1 year (more than 1 month)	180 (57.5%)
1 year or longer	33 (10.5%)
**Working currently as a clinical midwife**	240 (76.4%)
**Type of health facility working in**	
Primary care facility	146 (60.8%)
Hospital	86 (35.8%)
Other	8 (3.3%)
**Working in NGO-supported facility**	212 (88.7%)
**Employer (midwives)**	
Ministry of Health (MOH)	62 (25.8%)
NGO/Religious institution	159 (66.3%)
Private/self-employed	14 (5.8%)
Other	5 (2.1%)
**Location of work**	
Rural	114 (47.5%)
Town	94 (39.2%)
Juba	32 (13.3%)
Reasons for leaving previous jobs*	
Let go due to project closure or employer loss of funding	172 (29.3%)
Found a better job	99 (16.9%)
Low pay/not paid regularly	83 (14.1%)
Difficult work (e.g. colleagues, lack of supplies, too busy)	81 (13.8%)
Insecurity/felt unsafe	47 (8.0%)
Wanted to continue education	22 (3.7%)
Other	83 (14.1%)

*Participants could give multiple reasons and may have left multiple jobs; therefore, n > 314.

### Perceptions of the quality of their midwifery education

Overall, graduates reported the quality of education received was good or very good (100%) and that they felt well prepared to begin work as a midwife (99.4%). They most often reported clinical placements (60.1%) as the educational component that best prepared them for work, followed by classes (29.4%) and skill lab work (10.5%). The majority agreed or strongly agreed that they had sufficient opportunity to practise with anatomic models (80.5%), and that they had adequate supervision from tutors when performing on real patients during training (85.0%).

IDI respondents explained that their ability to apply their skills in practice demonstrated the strength of the education programme. They reiterated that the practical component was a vital part of the education because of the skills learned in this setting.

*“[The quality] was very good … First and foremost, the training was able to equip me with the skills, and those skills enable me actually to perform the midwifery work to the expectation. So, I can rate as very good because it has given me all the necessary skills that are needed to perform the duty of a midwife.”* (Male midwife 1)

Despite this strong support for the clinical practice, some IDI respondents reported difficulties practising on sufficient numbers of clients for some skills, especially at clinical sites that hosted students from multiple schools. They described a shortage of clients for specific obstetric complications, intrauterine device (IUD) insertion or removal, or manual vacuum aspiration (MVA) for post-abortion care. A few students reported challenges with mentorship, with too few tutors and high turnover in tutors to support their clinical practice.

*“Each and everyone was trying to get a patient. So, in that time I was a little bit worried. Why? Because patients are not enough.”* (Female midwife 2)

*“There was not sufficient time to practice all these things … . Some of the things were all pushed on the same semester, and you find there is pressure. So, we didn't really have enough time to practice what we have learnt.”* (Male midwife 1)

### Recommendations for strengthening the midwifery education programme

Graduates provided a wide range of recommendations to improve the midwifery education programme. They discussed needing a variety of resources, including additional textbooks, equipment for practising (such as anatomical models), and tutors. With respect to the practical component of their training, they cited a need for sufficient time to practise clinical skills and more opportunities to see a variety of complications, as well as improvements to the supervision and treatment of students during clinical attachment, including better mentorship.

*“There should be addition of staff, then also enough equipment should be there for the training for the practical parts and also enough books for students to do practice or to read on so that they get enough knowledge.”* (Female midwife 4)

*“I am suggesting also during the training or what they are doing, there should be proper supervision of the students because mostly when you find that when the students are not well supervised, when they go and they face challenges, they will not be able to practice what they are taught. Because you cannot learn something that you are not practiced in and you will not even continue doing it well.”* (Male midwife 3)

In addition to these suggestions to directly strengthen the education provided by these programmes, students also cited the need for resources to reduce stress on the students, such as living expenses.

*“And then also, we need also enough funds. You find somebody is studying and at the end of the day, somebody is also what? … Hustling to survive. So, how would you be able to balance between studying and working? You see? It is very hard.”* (Male midwife 1)

Graduates also offered recommendations to the government with respect to the need for sustained funding for the midwifery education programme.

*“There should be opening of more midwifery schools which are public, because sometimes going to a private school it might cost you a lot. We also have a few public health institutions and public universities in South Sudan, but they are not providing midwifery education, they don't have a midwifery program. So, we might … ask the Minister of Education to make sure that they incorporate midwifery education in those public universities so that somebody who is willing to study midwifery education, they are able to.”* (Male midwife 5)

### Frequency of SRH service provision and perceived competence

Graduates currently working as midwives were asked about how often they provided specific SRH services. Most reported providing antenatal care (87.5%), normal childbirth care (74.6%), and prevention of mother-to-child transmission of HIV (PMTCT) (57.5%) daily (Table 3). Midwives reported providing emergency obstetric and newborn care (EmONC) less frequently, with 15.8% providing this daily, and 33.8% providing it weekly. While 41.7% reported providing short-acting contraceptive methods daily, about a quarter reported providing long-acting reversible contraceptives (LARC) daily (25.0%) or weekly (27.9%). Clinical management of rape was the service they provided the least often, with 60.4% providing it rarely or never.

**Table 3.** Frequency of providing SRH services in current practice, Graduates currently working as midwives (n = 240)

	Daily n (%)	Weekly n (%)	Monthly n (%)	Rarely or never n (%)
Antenatal care (ANC)	210 (87.5%)	17 (7.1%)	8 (3.3%)	5 (2.1%)
Prevention of mother-to-child transmission of HIV (PMTCT)	138 (57.5%)	46 (19.2%)	27 (11.3%)	29 (12.1%)
Normal childbirth care	179 (74.6%)	42 (17.5%)	14 (5.8%)	5 (2.1%)
Emergency obstetric and newborn care (EmONC)	38 (15.8%)	81 (33.8%)	69 (28.7%)	52 (21.7%)
Short-acting contraceptive methods	131 (41.7%)	49 (15.6%)	38 (12.1%)	21 (6.7%)
Long-acting reversible contraception (implant, IUD)	60 (25.0%)	67 (27.9%)	50 (20.8%)	63 (26.3%)
Clinical management of rape	20 (8.3%)	26 (10.8%)	49 (20.4%)	145 (60.4%)

Several IDI respondents said that the lack of practice for some services reduced their feelings of competence. For example, limited numbers of clients seeking LARCs meant that many have never inserted or removed an IUD since graduation. Others referred to stockouts as the reason they were unable to practise PMTCT or post-abortion care.

Nearly all surveyed graduates reported feeling competent or very competent to provide the services which they practised most frequently, with over 84% reporting being very competent in providing antenatal care, safe childbirth care and short-acting contraceptive methods (Table 4). Over two-thirds reported feeling very competent to provide PMTCT (71.7%) and LARC (67.5%), while just over half (54.2%) reported being very competent to provide clinical management of rape. While few reported not feeling competent to provide any of these services, only 38% reported feeling very competent to provide EmONC, while 58.8% reported feeling competent.

**Table 4.** Perceived competency to provide SRH services, Graduates currently working as midwives (n = 240)

	Not competent^1^ n (%)	Competent^2^ n (%)	Very competent^3^ n (%)
Antenatal care	0	38 (15.8%)	202 (84.2%)
Prevention of mother-to-child transmission of HIV (PMTCT)	2 (0.8%)	66 (27.5%)	172 (71.7%)
Normal childbirth care	0	26 (10.8%)	214 (89.2%)
Emergency obstetric and newborn care (EmONC)	8 (3.3%)	141 (58.8%)	91 (37.9%)
Short-acting family planning methods	0	34 (14.2%)	206 (85.8%)
Long-acting reversible contraception (implant, IUD)	7 (2.9%)	71 (29.6%)	162 (67.5%)
Clinical management of rape	7 (2.9%)	102 (42.9%)	129 (54.2%)

^1^Not competent: may not result in desired outcome if performed without supervision.

^2^Competent: can perform safely and effectively, but would be more comfortable if supervised.

^3^Very competent: some expertise; can instruct others on how to perform task.

IDI respondents concurred, feeling confident to provide ANC, normal childbirth care, and short-acting contraception. Several reported feeling underprepared and wished for additional practice and training on some EmONC components (e.g. MVA), IUD insertion/removal, and care for survivors of gender-based violence due to low client load during their clinical training and in their current practice.

*“Let me say, when it comes to abnormal obstetrics, you know sometimes mothers will present with some complications … like abortions, obstructed labour, maybe premature fracture of membranes. Those are some of the complications [that] when a mother is presented with it … you feel like you are not comfortable.”* (Male midwife 6)

“*The areas [I am less comfortable with] are post abortion [care], like MVA, I cannot do it. In family planning, like this IUD, unless we are supported by another, how to insert, I cannot manage alone*.” (Female midwife 7)

Graduates described their training as equipping them with the skills they needed to fill a gap in health services in the communities they served. They referred to using the partograph to quickly identify problems during labour, managing or referring obstetric complications, and helping women to delay or space pregnancies with contraception. Several mentioned counselling adolescent girls to use contraception in order to continue in school. The midwives reported seeing increases in service utilisation, including in health facility births and contraceptive use, and decreases in maternal mortality.

“*Throughout my service, I am able to manage them [complications] and I am able to contribute to the progress of the health facility in meeting the objective of reducing maternal and infant mortality … meaning this is one of the strengths that the education has equipped me because I am able to manage all the problems*.” (Male midwife 5)

**Figure 1.**
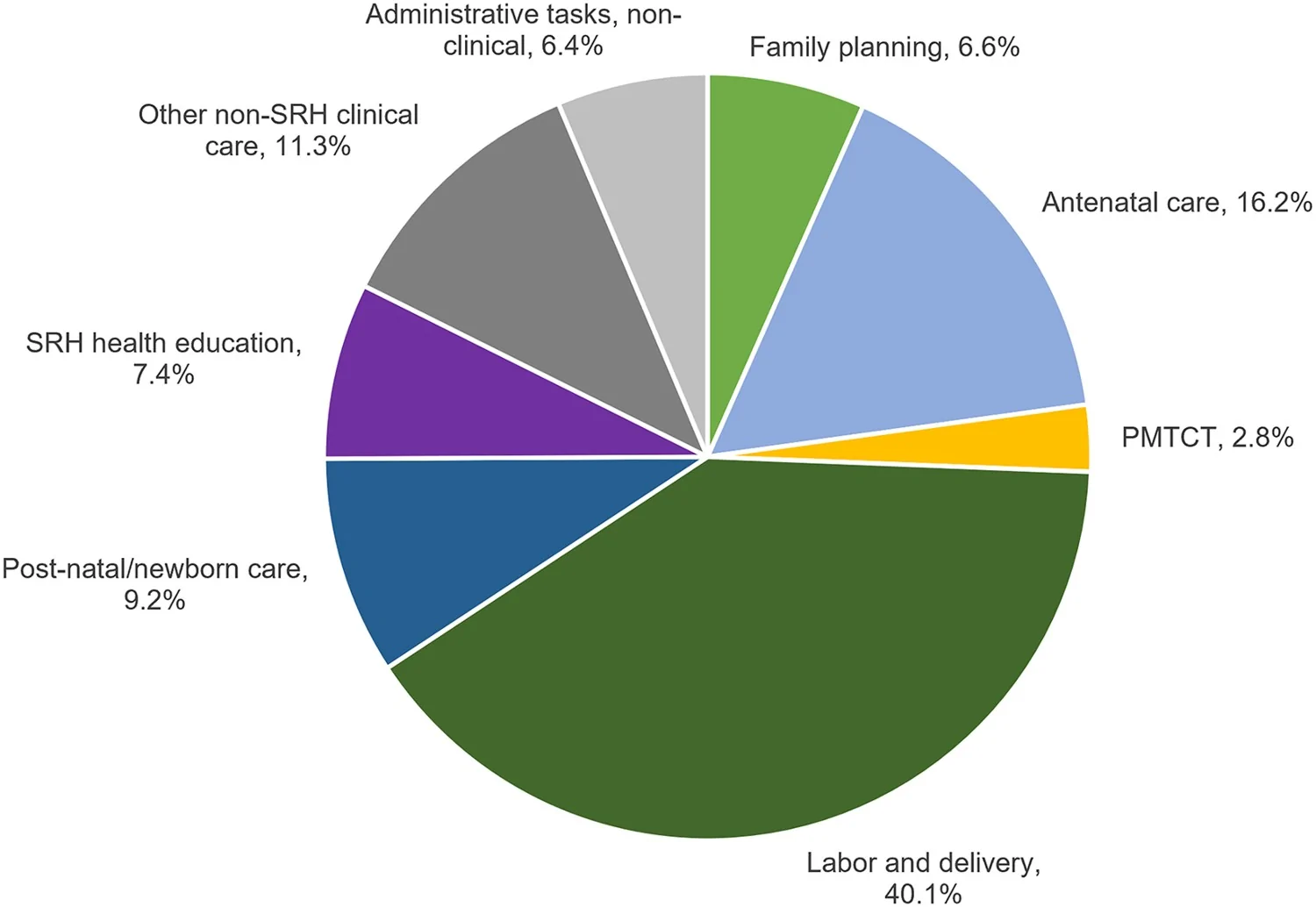
Percentage of time spent on services/tasks

Data for 274 total days of work were recorded in the clinical task diaries. Overall, midwives reported spending 82.3% of their time on SRH-specific tasks (Figure 1), with the most time spent on labour and childbirth (40.1%) followed by antenatal care (16.2%). Just over 10% of their time was spent on non-SRH services, like immunisation or outpatient care, and 6.5% of their time on administrative tasks.

### Main challenges in working as a midwife

The most commonly reported challenge midwives reported facing in their work since graduation was inadequate infrastructure and supplies (68.3%), followed by insecurity, including the lack of a safe place to live or poor accessibility to the health facility (45.8%) (Table 5). One-quarter reported lack of access to refresher training and limited access to guidelines or job aids. Another 25.8% reported challenges with clients, including low client numbers, language barriers, and lack of respect from clients.

**Table 5.** Main challenges in work, Graduates currently working as midwives (n = 240)

	n (%)
Lack of supplies, inadequate infrastructure	164 (68.3%)
Insecurity, poor accessibility, lack of safe accommodation	110 (45.8%)
Low client load, lack of respect from clients, language barriers	62 (25.8%)
Lack of refresher training, lack of job aids	61 (25.4%)
Difficult supervisor or colleagues, lack of respect from colleagues	20 (8.3%)
Other challenges	17 (7.1%)

IDI participants further explained how the lack of equipment and supplies, including medications, contraceptive methods and MVA kits, made their work more difficult. Several described needing to use the lights on their phones to manage births at night. These problems impacted their relationship with clients who were unhappy when asked to buy medication elsewhere or when they came to give birth in a dark health facility.

“*[Stockouts] are one of the barriers like sometimes we don’t have antibiotics. So, it’s a problem. Even now we do not have anything for a person who is having eclampsia and anti-convulsant medicine*.” (Female midwife 8)

They also described insufficient staff at the health facility, and the burden this placed on them to meet the community’s needs, often as the only qualified midwife working alongside traditional birth attendants with no formal training.

“*Another thing is the number of staff, especially where I am working, it is only me alone who is working here. So, when I am not there, then things will stop – there is nothing that will work because there is nobody who can make the record. These people here, they don’t know how to write, so when I go for a workshop, things here also close. So, these are the challenges that do not allow services to go smoothly for the people*.” (Male midwife 9)

Insufficient and delayed remuneration was a common challenge that midwives mentioned, particularly those employed by the MOH. For example, some midwives reported working as volunteers at health facilities for a period of time before finally finding a paid position.

“*Salary is the problem also because the work we are doing, of course is not for salary. If we were to say that this salary is not enough, then I would have to leave this job. But it’s because I am caring for the community and above all, I was trained to do it. So, I will continue to do it, but the salary actually does not match with the [work] we are doing, the work is too much. But it is not because of salary. If it is because of salary I can go to do my private business*.” (Male midwife 10)

“*My salary … even though it’s too small because I am saving the life of people, because I like my work, even though up to now I’m still volunteer not staff here*.” (Female midwife 7)

Despite the commitment expressed by some midwives to continue doing this important work, others expressed concern that they may need to change careers, or at least leave government positions, in favour of NGO-funded ones, for example, in order to make a living.

“*The issue of salary is a challenge, the little money given to us cannot do anything … that is why you see many midwives have moved away from the government … Even me if I get a chance of getting another job different from the midwifery duty then I will also go*.” (Male midwife 9)

Less than 10% of survey respondents reported difficulties with colleagues, similar to the IDI participants. Overall, they described mostly positive work environments in which their supervisors and colleagues were supportive and provided recognition for their work.

“*My superior has been so collaborative with me, and I can say this is the environment I enjoy working in most of the time because the supervisor is very cooperative. And then we always have case sharing experience and knowledge sharing … My colleague also, we have enjoyed a very good partnership and we have been of help to each other, and whenever I need help she always comes in and whenever she needs [something] the reverse is always true*.” (Male midwife 11)

### Impact of insecurity

Insecurity came up often in the IDIs, with several describing times when conflict temporarily interrupted their studies. Graduates of KKHSI, in particular, described the impact of insecurity on their education, including that the curriculum was not implemented as designed, rushing the learning process. In addition, class size was mentioned as a challenge after the school moved to share JCONAM’s space in Juba in 2016 due to insecurity in Kajo Keji.

“*One of the other weaknesses of education here in South Sudan, is also the insecurity issues. It is making the education very difficult because things may not go the way it has been planned. Sometimes your mind is here for studies sometimes also some other problems arise due to insecurity, and it becomes very hard for the students really to put their mind and focus on what they are supposed to learn*.” (Male midwife 1)

“*When we were in Juba, we were put in one place … We were about 70 something [students] there, so we were packed in one place, so learning became so difficult for some of us … it became very hard sometimes because we’re combined in one class where the number was large*.” (Male midwife 3)

Some graduates recommended the government address insecurity to improve education.

“*We need proper security. The situation of the security must be normal for the training in South Sudan to go normal because you cannot be able to learn and acquire necessary information, to learn certain skills when your heart is not safe, or when you don't feel safe in the place. Your mind is thinking of the situation … So, improving the security can improve the education in South Sudan*.” (Male midwife 1)

Insecurity was also a stress-inducing challenge to midwives’ current work, including difficulties finding safe accommodation near the health facilities. IDI respondents reported lost belongings, sleeping at the hospital or sheltering at home during periodic bouts of conflict; others described difficulties communicating with or reaching family. Respondents described how insecurity disrupted access to the health facility for them and their clients and prevented many from offering night-time births, leaving the women with no option but to give birth at home.

“*In my first placement where I was assigned was a very [remote] place. Then the conflict happened there … One good thing, they took us for a training [meaning they were absent when the conflict started] … Then, the [conflict] happened - everything was destroyed, even the medical services there, they were all destroyed including our belongings because everything was destroyed*.” (Male midwife 10)

“*So [when we were not allowed to go to the health facility], I was not happy because there are a lot of pregnant mothers who were suffering … They need our services, but we were not there. … But now I’m also grateful because they called us back, at least now we are helping the pregnant mothers. Always they are the very people who are affected most*.” (Female midwife 2)

### Advice for future midwifery students

Finally, graduates offered advice to current and future midwifery students. They expressed the need for students to be dedicated to the profession, to pay attention during coursework and training. In addition, the importance of clinical practice during training was emphasised. Teamwork, humility, communication, and flexibility were traits midwives identified as important for future colleagues.

“*If they are still in their school, they should cooperate with their teachers. When they were told to do something, they have to understand because the same thing we are learning here is the same thing you will transfer to the facility, to the hospital and you will be able to help the community*.” (Female midwife 12)

“*Again, they are able to understand that the community we serve, especially South Sudan, generally, it has a diverse community. So, you have to know the community, you have to understand the community you serve. So, meaning you need to understand the background of the community very well, so that you are able to build a good relationship with them. Then students need to have good communication skills – this is something that is very important*.” (Male midwife 5)

### Pride and satisfaction in being a midwife

Despite the numerous challenges and concerns, every IDI respondent reported pride in their job and their role as a midwife. A source of their pride came from the fact that the services they provide are important to the communities, which were largely receptive and appreciative of their work. Several commented that the community didn’t have those services before they arrived, further suggesting that this midwifery education programme has increased access to skilled midwifery care throughout the country. They described caring for their clients and their community, as well as the respect and recognition they in turn receive in the community.

“*I am proud to be a midwife because I save a lot of lives*.” (Female midwife 13)

“*In general, I feel proud of being a midwife. … And also, I am proud that the community is able to appreciate my being with them and giving them what they have been missing for so long*.” (Male midwife 5)

## Discussion

Midwives play a critical role in reducing preventable maternal deaths and disability worldwide, particularly in low- and middle-income countries where health facilities are few and far between.^15,28^ Our study found that graduates of the IMC-supported education programme were applying the skills they were trained in, with most currently working as midwives and actively delivering SRH services. This study is one of the few to document the perspectives of midwifery graduates in a conflict-affected setting, providing insights into how competency-based training translates into practice in low-resource and insecure environments. The midwifery education programme has expanded the reach of SRH services across South Sudan, with graduates working throughout the country, nearly half in rural areas where the need for skilled health workers is greatest. By providing essential care during pregnancy and childbirth, despite limited infrastructure, insecurity, and other resource constraints, these graduate midwives are contributing to improving maternal health in South Sudan, particularly among those who might otherwise face life-threatening complications without skilled support.

Overall, the graduates were satisfied with the education they received, reporting it was of high quality and prepared them well to begin work as midwives. While more than 80% highly rated their clinical practice and tutors, they also highlighted some areas of the education programme that could be strengthened. For example, some mentioned the need for enhanced supervision during clinical practice, including better-trained tutors or less turnover. The insufficient practice some students reported, due, in part, to overcrowding in clinical sites, is sometimes seen in resource-limited settings.^29,30^ However, the importance of competency-based training provided by trained tutors and preceptors cannot be understated.^31^ In findings we reported elsewhere, key stakeholders called for increased and sustained government funding for the midwifery education programme as well as health services in response to these issues reported by the graduates.^24^ Investment in midwifery education is critical to ensure midwives are well-skilled to provide the full range of midwifery services.^29^

Unsurprisingly, the services for which graduates reported less competence were also the ones they provided less frequently, in particular EmONC, LARC, and clinical management of rape. Graduates attributed this to several factors, including low client load during clinical placements or in their current work, as well as stockouts or lack of equipment. Feeling inadequately prepared to provide a service makes them less likely to offer it.^32,33^ For example, a midwife who lacks confidence in their IUD insertion skills is unlikely to offer IUDs as a potential option during contraceptive counselling, resulting in few clients who request one, which will further weaken the midwife’s skills. It is critical that students receive adequate practice for all competencies during their training so that women have access to the full range of SRH services midwives should provide. To address limited clinical practice in conflict-affected, low-volume settings, education programmes and training sites should adjust supervision and rotations to ensure students receive sufficient hands-on practice, including organising shifts to minimise overcrowding and providing additional lab practice on anatomic models for services with few clients. After deployment, MOH supervisors and partners can support skill retention through supervision, simulation, and periodic rotations to facilities with higher client load, including teaching hospitals.

The lack of practice due to insufficient supplies and equipment in their current health facilities further erodes graduate midwives’ competency for services they cannot practise. Inadequate supplies and infrastructure were the most commonly reported challenge in graduates’ current work, including stockouts and lack of light for night-time births. Not only do supply problems negatively impact midwives’ competency, but they also impact their relationship with clients by increasing frustration with midwives’ inability to provide good quality care and clients consequently choosing not to visit the facility.^34^ Improving and maintaining competence for all midwifery skills is especially challenging in resource-poor conflict-affected countries like South Sudan.^15^ It is, therefore, critical for the MOH, their NGO partners and facility supervisors to take the lead in strengthening midwives’ support systems, including the integration of refresher training, supportive supervision, and mentorship into health programming, to support midwives to provide good quality maternal and newborn care.^15,35,36^

Midwives, especially those in rural locations, may be the only midwives available, resulting in a lack of mentorship but also overwork. Decades of war have affected South Sudan’s investment in human resources for health. Although the density of nursing and midwifery personnel increased from 3.7 per 10,000 population in 2018 to 6.9 in 2022, it remains well below WHO’s recommended threshold^30^ and that of neighbouring countries, Kenya (20.2) and Uganda (22.6), highlighting both South Sudan’s progress and ongoing critical gaps.^37,38^ Insecurity can further reinforce this isolation by preventing supportive supervision visits. In addition, health systems issues like lack of supplies and insufficient personnel require midwives to adapt, which may increase instances of mistreatment or disrespect of women during pregnancy and birth.^39^ Several graduates reported having only unskilled traditional birth attendants (TBA) supporting them in the maternity unit, most of whom were illiterate and unable to assist with documentation and record-keeping. Although some global efforts to integrate TBAs into the formal health system have yielded mixed outcomes,^40^ evidence shows that TBAs alone do not reduce maternal mortality.^41^

Graduates described insecurity as a major factor affecting both their education and their work, and as a reason for leaving a job. It was the second most frequently mentioned challenge, consistent with findings in other humanitarian settings, including South Sudan and Somalia.^22,42,43^ Insecurity disrupted their education, causing temporary school closures and leading to overcrowding in JCONAM when students from KKHSI were relocated after their school was destroyed. Conflict also affected their ability to find safe housing near their place of work, their own and their clients’ ability to reach the health facility, and prevented them from managing night-time births – one reason often given by women for giving birth at home.^3^

Insufficient remuneration was another challenge frequently mentioned by graduates, and was a reason for leaving a job, similar to other humanitarian settings.^15^ Low salaries were cited as a source of frustration, with some needing to “do business” on the side to make ends meet. Several reported working as volunteers before gaining a paid position. Although some mentioned they were considering leaving the profession in order to support their family, others reiterated their commitment to their work. Insufficient remuneration is linked to poor motivation and performance in other low-income countries,^33^ and results in health workers leaving the public sector. The MOH should improve its plan for the deployment of midwives, including sufficient salary to meet their basic needs.

Globally, the midwifery profession is dominated by women; however, 41% of the midwife graduates in our survey were male. In a country where only 11% of women complete secondary education,^44^ a criterion for enrolment in the midwifery programme, training male midwives can help increase the number of midwives in the country. Colleagues at SSNAMA suggested that their initiative to provide career guidance to both young women and men contributed to increased male enrolment in midwifery schools.^45^ In a related article, we noted that the women in the community initially expressed surprise at being cared for by a male midwife, but mostly came to appreciate them, with a few exceptions.^24^ The male midwives concurred, reporting some initial resistance, but said that they were largely accepted. Similar mixed reactions were observed in another study in South Sudan, with some women who had been cared for by both male and female midwives finding the men more considerate of them during birth while those who had not yet met a male midwife expressed discomfort with the idea.^46^ SSNAMA colleagues, based on their own assessments, reported that some women preferred a male midwife, and that growing interest among South Sudanese men in pursuing education and assisting their communities likely contributed to the rise in male midwifery students. Studies from other contexts suggest that while women often express a preference for female midwives during childbirth, male midwives are generally accepted and do not negatively impact labour and birth experiences.^47,48^ These findings suggest that in some contexts, male midwives can help to address gaps in female education and challenge traditional gender norms.

Despite the challenges graduates faced during their work, they reported feeling pride and satisfaction in the vital role they play as midwives in extending equitable health coverage in South Sudan, which is consistent with other studies.^49–51^ These findings highlight the resilience and dedication of midwives working under challenging conditions and their critical role in sustaining maternal and newborn health in fragile settings. For example, professional pride among midwives in the Democratic Republic of the Congo resulted in feelings of commitment, duty, and resilience, despite their difficult working conditions,^51^ similar to our findings. The graduates in our study also praised the training programme for providing them with the skills they needed to fill critical health service gaps in the communities they served. The communities, in turn, have been receptive and appreciative of their work,^24^ with graduates observing increased service utilisation and a decrease in maternal mortality. A community’s recognition of a midwife’s work is important and has been shown to increase job satisfaction and motivation to practice midwifery.^33^

### Limitations

While efforts were made to reach all graduates of the three schools for the survey, some were unreachable. It is likely that those currently working in rural areas were underrepresented, as they were more likely to lack mobile phone service. While the surveys were conducted in English, the language of instruction at the midwifery schools, poor phone connections may have limited understanding of some survey questions. IDIs were conducted in person with a convenience sample of midwives who worked in or around Juba, Malakal or Wau for security and accessibility reasons. This resulted in a group who were less diverse than intended and meant the views of those working in more remote or unsafe locations were underrepresented.

## Conclusions

Midwives are essential to achieving the United Nations Sustainable Development Goals (SDGs), particularly the target for reducing maternal and newborn mortality. While multiple factors likely contributed to the decline in maternal mortality in South Sudan, the nearly 500 midwives trained by the IMC midwifery education programme have been a significant driver of this progress in a fragile context. Despite the weaknesses of South Sudan’s health system, these midwives reported high overall satisfaction with their training, a strong sense of preparedness for their roles, and deep pride in their profession. They serve throughout the country, including many in rural areas, supporting women through safe and healthy pregnancies and empowering women and couples to exercise their reproductive rights. These findings demonstrate how competency-based education programmes can strengthen the midwifery workforce in fragile settings and expand access to essential SRH services.

To sustain these gains, continued investment and collaboration among the MOH, NGO partners, and donors must support midwives’ professional development ensuing they remain aligned with evolving global standards and emerging health needs and expand employment opportunities. Attention must also address persistent workforce challenges, including insecurity, inadequate remuneration, and shortages of essential medicines, equipment, and supplies required for quality care. Without focused and coordinated action, South Sudan risks losing the progress made in strengthening its midwifery workforce, improving maternal and newborn health outcomes, and advancing women’s access to good quality, respectful SRH care.

## Supplementary Material

Supplemental File 1. Midwifery evaluation study tools

## Data Availability

Relevant survey data are available on Academic Commons, Columbia University’s online research repository (10.7916/d39h-za86). The qualitative data used during the study are available from the corresponding author on reasonable request.
