# Supplementary material for: “I am proud to be a midwife because I save a lot of lives”: survey and interviews with graduates of International Medical Corps’ midwifery education programme in South Sudan
Source: Sex Reprod Health Matters. 2026 Jul 31;33(1):2694248. doi: 10.1080/26410397.2026.2694248 (PMC13573500; doi:10.1080/26410397.2026.2694248)
Supplement: Supplementary file 1 [file ZRHM_A_2694248_SM9723.pdf]

Survey of midwifery school graduates, South Sudan

IDNO:
Date:
Interviewer:

No.	Questions and filters	Coding categories	Skip to
Q101	What midwifery diploma program (highest level) did you complete?<br>[If you did more than one midwifery degree, please put the highest level degree obtained.]	1 Registered midwife<br>2 Enrolled (certified) midwife<br>3 Community midwife<br>4 Other (specify)____	
Q102	Which midwifery school did you graduate from with that degree?<br>[highest degree]	1 Juba College of Nursing and Midwifery (JCONAM)<br>2 Wau Health Sciences Institute (WHSI)<br>3 Kajo Keji Health Sciences Institute (KKHSI)	
Q103	What year did you graduate from midwifery school?<br>[If you did more than one midwifery degree, please put year obtained highest level degree.]	___ ____ ___ ___<br><br>(KOBO Limit years **2013 – 2021)**	
Q104	Did you receive financial support (scholarship) from International Medical Corps (IMC) for your midwifery degree?	1 Yes<br>0 No	
Q105	Are you currently working clinically as a midwife?	1 Yes<br>0 No	→**Q108**
Q106	What type of health facility do you work in?	1. PHCU<br>2. PHCC<br>3. Hospital<br>4. Private health facility<br>5. IDP/ POC / Refugee Camp Facility<br>6. Other (Specify)____	
Q107	Is this health facility supported by an NGO?	1 Yes<br>0 No	Any answer go to Q110
Q108	What is your current job?<br><br>** [Q108-Q109 are for those whom Q105=0.]*	1. Other type of clinician<br>2. Non-clinical work at NGO<br>3. Non-clinical work at MOH/Government<br>4. Police or Military forces<br>5. Private business<br>6. Current student<br>7. Unemployed<br>8. Other (specify)___	→**Q113**
Q109	Is your current job related to reproductive health or maternal health?	1 Yes<br>0 No	
Q110	Who is your employer?<br><br>**[Q110-Q112 should be for all except Q108=7.]*	1 Ministry of Health<br>2 International NGO<br>3 National (South Sudanese) NGO<br>4 Private company<br>5 Self-employed<br>6 Student<br>7 Other (specify)___	

No.	Questions and filters	Coding categories	Skip to
Q111	In which state are you currently working or living?	1. Abyei<br>2. Central Equatoria<br>3. Eastern Equatoria<br>4. Jonglei<br>5. Lakes<br>6. Northen Bahr El Ghazal<br>7. Pibor<br>8. Ruweng<br>9. Unity<br>10. Upper Nile<br>11. Warrap<br>12. Western Bahr Al Ghazal<br>13. Western Equatoria<br>14. Outside of South Sudan (specify country)_	
Q112	Are you currently working in a rural location, a town or in Juba?<br>*[If response is POC, ask if POC located in urban or rural area]*	1 Rural<br>2 Town<br>3 City (Juba)	**Any answer Q114**
Q113	Are you looking for work?	1 Yes<br>0 No	
Q114	How many different job postings (of 6 months or longer) before this job have you had since you graduated?	__ __<br>(KOBO limit 0-20)	
Q115	What was the job you held before this one?<br><br>[*Ask Qs 115-117 for each number of jobs mentioned in Q114,* for a maximum of 3 jobs]	1 Working clinically as a midwife in a health facility<br>2 Non-clinical work at NGO<br>3 Non-clinical work at MOH<br>4 Police or Military forces<br>5 Private business<br>6 Current student<br>7 Unemployed<br>8 Other (specify)___	
Q116	Who was your employer?	1 Ministry of Health/Government of South Sudan<br>2 International NGO<br>3 National NGO<br>4 Private company<br>5 Self-employed<br>6 Student<br>7 Other (specify)___	

No.	Questions and filters	Coding categories	Skip to
Q117	What is the main reason you left this job?<br><br>[Limit to top 3 reasons]	1. Found a better job<br>2. Wanted to be closer to family<br>3. Let go due to project closure or employer loss of funding<br>4. Difficulties with supervisor/colleagues<br>5. Lack of motivation/didn't like job<br>6. Too difficult (lack supplies, staff, too busy, etc)<br>7. Low pay / not paid regularly<br>8. Wanted to continue my education<br>9. Wanted to start private business<br>10. Insecurity / felt unsafe working there<br>11. Got married / pregnant / had child<br>12. Sickness<br>13. Other (Specify)____<br>99. Prefer not to answer	
**IF Q105=0 (NOT working as midwife): go to Q140 Challenges**<br>**IF Q105=1 (currently working as midwife):** I'd now like to ask about the midwifery services you provide in your current job			
Q118	How frequently do you provide antenatal care?	4 Daily (at least once per day)<br>3 Weekly (at least once per week but not daily)<br>2 Monthly (at least once per month but not weekly)<br>1 Rarely (less than once per month)<br>0 Never (not performed due to lack of capacity or opportunity)	
Q119	Where have you received training to provide antenatal care?<br><br>[PROBE:] Anywhere else?	SELECT MULTIPLE<br>1 Pre-service education (Midwifery school)<br>2 In-service training (formal training workshop, refresher training)<br>3 On-the-job training (informal training in health facility from coworkers or supervisor)<br>4 Not trained (no formal or informal training)	
Q120	How well do you feel you are able to provide antenatal care?	3 Very competent (some expertise; can instruct others on how to perform task)<br>2 Competent (can perform safely and effectively, but would be more comfortable if supervised)<br>1 Not competent (may not result in desired outcome if performed without supervision)	
Q121	How frequently do you help pregnant women deliver (provide normal delivery care)?	4 Daily (at least once per day)<br>3 Weekly (at least once per week but not daily)<br>2 Monthly (at least once per month but not weekly)<br>1 Rarely (less than once per month)<br>0 Never (not performed due to lack of capacity or opportunity)	

No.	Questions and filters	Coding categories	Skip to
Q122	Where have you received training to provide normal delivery care?<br><br>[PROBE:] Anywhere else?	SELECT MULTIPLE<br>1 Pre-service education (Midwifery school)<br>2 In-service training (formal training workshop, refresher training)<br>3 On-the-job training (informal training in health facility from coworkers or supervisor)<br>**4 Not trained (no formal or informal training)**	
Q123	How well do you feel you are able to provide normal delivery care?	3 Very competent (some expertise; can instruct others on how to perform task)<br>2 Competent (can perform safely and effectively, but would be more comfortable if supervised)<br>1 Not competent (may not result in desired outcome if performed without supervision)	
Q124	How frequently do you manage complicated deliveries / provide EmONC, emergency obstetric and newborn care (administration of parenteral antibiotics, anticonvulsants or uterotonics, manual removal of placenta, removal of retained products, newborn resuscitation, blood transfusion)?	4 Daily (at least once per day)<br>3 Weekly (at least once per week but not daily)<br>2 Monthly (at least once per month but not weekly)<br>1 Rarely (less than once per month)<br>0 Never (not performed due to lack of capacity or opportunity)	
Q125	Where have you received training to manage complicated deliveries / provide EmONC (emergency obstetric and newborn care)?<br><br>[PROBE:] Anywhere else?	SELECT MULTIPLE<br>1 Pre-service education (Midwifery school)<br>2 In-service training (formal training workshop, refresher training)<br>3 On-the-job training (informal training in health facility from coworkers or supervisor)<br>**4 Not trained (no formal or informal training)**	
Q126	How well do you feel you are able to manage complicated deliveries / provide EmONC?	3 Very competent (some expertise; can instruct others on how to perform task)<br>2 Competent (can perform safely and effectively, but would be more comfortable if supervised)<br>1 Not competent (may not result in desired outcome if performed without supervision)	
Q127	How frequently do you provide short-acting methods of family planning (pills, injectables, condoms)?	4 Daily (at least once per day)<br>3 Weekly (at least once per week but not daily)<br>2 Monthly (at least once per month but not weekly)<br>1 Rarely (less than once per month)<br>0 Never (not performed due to lack of capacity or opportunity)	
Q128	Where have you received training to provide short-acting family planning methods?<br><br>[PROBE:] Anywhere else?	SELECT MULTIPLE<br>1 Pre-service education (Midwifery school)<br>2 In-service training (formal training workshop, refresher training)<br>3 On-the-job training (informal training in health facility from coworkers or supervisor)<br>**4 Not trained (no formal or informal training)**	

No.	Questions and filters	Coding categories	Skip to
Q129	How well do you feel you are able to provide short-acting family planning methods?	3 Very competent (some expertise; can instruct others on how to perform task)<br>2 Competent (can perform safely and effectively, but would be more comfortable if supervised)<br>1 Not competent (may not result in desired outcome if performed without supervision)	
Q130	How frequently do you provide long-acting methods of family planning (implants, IUDs)?	4 Daily (at least once per day)<br>3 Weekly (at least once per week but not daily)<br>2 Monthly (at least once per month but not weekly)<br>1 Rarely (less than once per month)<br>0 Never (not performed due to lack of capacity or opportunity)	
Q131	Where have you received training to provide implants and IUDs?<br><br>[PROBE:] Anywhere else?	SELECT MULTIPLE<br>1 Pre-service education (Midwifery school)<br>2 In-service training (formal training workshop, refresher training)<br>3 On-the-job training (informal training in health facility from coworkers or supervisor)<br>**4 Not trained (no formal or informal training)**	
Q132	How well do you feel you are able to provide implants and IUDs?	3 Very competent (some expertise; can instruct others on how to perform task)<br>2 Competent (can perform safely and effectively, but would be more comfortable if supervised)<br>1 Not competent (may not result in desired outcome if performed without supervision)	
Q133	How frequently do you provide clinical management of rape?	4 Daily (at least once per day)<br>3 Weekly (at least once per week but not daily)<br>2 Monthly (at least once per month but not weekly)<br>1 Rarely (less than once per month)<br>0 Never (not performed due to lack of capacity or opportunity)	
Q134	Where have you received training to provide clinical management of rape?<br><br>[PROBE:] Anywhere else?	SELECT MULTIPLE<br>1 Pre-service education (Midwifery school)<br>2 In-service training (formal training workshop, refresher training)<br>3 On-the-job training (informal training in health facility from coworkers or supervisor)<br>**4 Not trained (no formal or informal training)**	
Q135	How well do you feel you are able to provide clinical management of rape?	3 Very competent (some expertise; can instruct others on how to perform task)<br>2 Competent (can perform safely and effectively, but would be more comfortable if supervised)<br>1 Not competent (may not result in desired outcome if performed without supervision)	

No.	Questions and filters	Coding categories	Skip to
Q136	How frequently do you provide prevention of mother to child transmission of HIV (PMTCT)?	4 Daily (at least once per day)<br>3 Weekly (at least once per week but not daily)<br>2 Monthly (at least once per month but not weekly)<br>1 Rarely (less than once per month)<br>0 Never (not performed due to lack of capacity or opportunity)	
Q137	Where have you received training to provide prevention of mother to child transmission of HIV (PMTCT)?<br><br>[PROBE:] Anywhere else?	SELECT MULTIPLE<br>1 Pre-service education (Midwifery school)<br>2 In-service training (formal training workshop, refresher training)<br>3 On-the-job training (informal training in health facility from coworkers or supervisor)<br>**4 Not trained (no formal or informal training)**	
Q138	How well do you feel you are able to provide prevention of mother to child transmission of HIV (PMTCT)?	3 Very competent (some expertise; can instruct others on how to perform task)<br>2 Competent (can perform safely and effectively, but would be more comfortable if supervised)<br>1 Not competent (may not result in desired outcome if performed without supervision)	
Q139	What are the main challenges you have faced in your midwifery work since graduation?	**SELECT MULTIPLE**<br>1. Lack of supplies and equipment<br>2. Inadequate health facility infrastructure<br>3. Too few clients/patients<br>4. Lack of respect at work (from colleagues/ supervisors)<br>5. Lack of respect from clients<br>6. Lack of opportunities for refresher trainings<br>7. Limited or no access to guidelines and job-aids<br>8. Difficult supervisors/colleagues<br>9. Insufficient number of staff (too busy)<br>10. Appropriate salary/ compensation<br>11. Finding a job<br>12. Insecurity<br>13. Poor accessibility to health facility (due to poor/no road, flood)<br>14. Finding a safe/clean place to live near facility<br>15. Political pressure<br>16. Other (specify)	
Please think about the education you received at the midwifery school to answer the next questions			
Q140	What do you think of the quality of the education that you received at the midwifery school overall? Was the quality Very good, Good, Acceptable, Poor or Very poor?	5 Very good<br>4 Good<br>3 Acceptable<br>2 Poor<br>1 Very poor	
Q141	When you graduated from midwifery, how well prepared did you feel for beginning work as a midwife?	3 Well prepared<br>2 Unsure of how well prepared<br>1 Did not feel well prepared	

No.	Questions and filters	Coding categories	Skip to
Q142	Which element of your midwifery education do you believe best prepared you for work as a midwife: classes, skill lab work or clinical placements??	1 Classes<br>2 Skill lab work<br>3 Clinical placements	
Q143	Which services do you wish you had received more practice/training on?	SELECT MULTIPLE<br>1 Antenatal care<br>2 Normal delivery care<br>3 Emergency obstetric care<br>4 Emergency newborn care<br>5 Postpartum care (mother)<br>6 Postnatal care (infant)<br>7 Short-acting family planning methods<br>8 Long-acting family planning methods<br>9 Post abortion care<br>10 prevention of mother to child transmission of HIV (PMTCT)<br>11 clinical management of rape<br>12 Other (specify)	
Now I am going to read you a few statements. For each statement, I would like you to tell me if you strongly disagree, if you disagree, if you agree or if you strongly agree with the phrase.			
Q144	You had opportunities to practice (most skills) with anatomic models before you were expected to perform them with real patients.	1 Strongly disagree<br>2 Disagree<br>3 Agree<br>4 Strongly agree	
Q145	You had adequate supervision from tutors when you performed on real patients during your practical training.	1 Strongly disagree<br>2 Disagree<br>3 Agree<br>4 Strongly agree	
Q146	Thinking back to when you graduated with your midwifery degree, how long after graduation did you start a new job?	1 Immediately/less than 1 month<br>2 1-6 months<br>3 7-11 months (more than 6 months, less than 1 year)<br>4 1-2 years<br>5 More than 2 years	
Q147	Have you registered with the Nurses and Midwives Council?	1 Yes<br>0 No<br>8 Not sure	
Q148	Are you a member of South Sudan Nurses and Midwives Association (SSNAMA)?	1 Yes<br>0 No<br>8 Not sure	
**Now I'd like to ask a few questions about you:**			
Q149	In what year were you born?	Year [__|__|__|__]<br>Don't Know 8888	
Q150	What is your gender?	1 Female<br>2 Male	

No.	Questions and filters	Coding categories	Skip to
Q151	What state are you from?	1. Abyei<br>2. Central Equatoria<br>3. Eastern Equatoria<br>4. Jonglei<br>5. Lakes<br>6. Northen Bahr El Ghazal<br>7. Pibor<br>8. Ruweng<br>9. Unity<br>10. Upper Nile<br>11. Warrap<br>12. Western Bahr Al Ghazal<br>13. Western Equatoria	
Q152	What is your current marital status?	0 Never married<br>1 Currently married or living with someone as if married<br>2 Divorced or separated<br>3 Widowed<br>9 Prefer not to answer	
Q153	Do you have any children? How many?	— —<br>[KOBO limit 0-12]	
Q154	Are you currently displaced from your home?	0 No<br>1 Yes, I am an IDP (in South Sudan)<br>2 Yes, I am a refugee (outside South Sudan)<br>9 Prefer not to answer	

IDI Guide for Graduate Midwives

Respondent information

Please introduce yourself.
How old are you?
Which midwifery diploma do you have: Enrolled midwife / Registered midwife?
Which school did you attend? When did you graduate?
Are you registered with Nurses and Midwives Council?
Are you a member of South Sudan Nurses and Midwives Association (SSNAMA)?
What is the name of this health facility?
What level facility is this? (ex: hospital, PHCU, PHCC, etc):
Does this facility receive NGO support?

Effectiveness of education program to prepare students for practice

1. What do you think of the quality of the education/training that you received? Why do you say that?
Probe: Do you think the quality was very poor, acceptable, or very good? Why?

2. What are the strengths of the education that you received? Were there any different or new approaches or good practices used during your training? What are they?

3. What are the weaknesses of the education that you received? Why?

4. Would you please tell me about the practical clinical training you received? Was it adequate? Insufficient? Why?
Probe: Were there enough patients to practice on? Did you get sufficient practice for all services?

5. Think back to when you graduated, what were the midwifery services that you felt most comfortable with? Which areas did you think you were best prepared for?
Probe: How did you feel about your skills to provide EmONC? Family Planning? What about long-acting methods? Post-abortion care (PAC)?

6. Think back to when you graduated, what were the midwifery services that you felt the least comfortable with? Which areas did you wish you had received more training on? Why?
Probe: How would you advise to improve the program to help new students become stronger in these areas?

7. Overall, how prepared did you feel to take care of women (and newborns) when you graduated? If no, why not?

8. What is the most important thing you think could be improved to make new graduates feel even better prepared?

9. Thinking about your work environment, have you received any refresher training since you graduated? Who provided this training? What topics were covered? Do you wish to receive more refresher training? On what topics?

10. How have you been treated by your supervisors? By your colleagues? Do you feel supported in your work? Do they recognize your skills? How could this be improved?

Impact

11. Overall, how has been your experience working as a midwife?
Probe: How is your reception by the community? What do your family and spouse think about your profession? Insecurity? Impact on personal life, etc

12. How has the insecurity affected your work?
Probe: Does it impact what you do? When you work?

13. How are you perceived/treated in the community? By women? By men? By community leaders?

14. Has the education you received made a difference for the women in the community that you serve? Did it have any impact on others as well?

15. Were there any surprises or unintended/unexpected results? What were they?

16. Are the services you were trained to provide relevant and appropriate for the needs of people in your community? Why or why not?

17. Are there people who do not benefit from your services? Who are they? Why do they not use your services?

Barriers to ability to deliver quality reproductive health care

18. What are the barriers for you to deliver the best possible care to your patients and their families?
Probe: medical supplies/equipment; knowledge; experience; support by a medical team (e.g., doctor, surgeon, senior midwives), difficulty in referring, transport, communication, cost, insecurity, etc.

19. Can you suggest ways that we can help reduce these barriers?

Lessons learned

20. What do you feel most proud about?

21. What would you like to tell new midwife students that would help them succeed in the training program? To succeed in working as a midwife?

22. Do you think you will continue working as a midwife? Why or why not? (Probe: salary)

23. What recommendations do you have to improve the training program?

24. Do you have anything else you would like to share or suggest?
